# 4,5-Bis(1*H*-tetra­zol-5-yl)-1*H*-imidazole monohydrate

**DOI:** 10.1107/S1600536809017899

**Published:** 2009-05-29

**Authors:** Min Guo

**Affiliations:** a Ordered Matter Science Research Center, Southeast University, Nanjing 210096, People’s Republic of China

## Abstract

The title compound, C_5_H_4_N_10_·H_2_O, is composed of three five-membered rings that are essentially coplanar, the dihedral angles between the imidazole ring and the tetra­zole rings being 3.5 (2) and 3.0 (2)°. In the crystal, inter­molecular O—H⋯N, N—H⋯O and N—H⋯N hydrogen bonds lead to the formation of a three-dimensional network. An intra­molecular N—H⋯N hydrogen bond is also present.

## Related literature

For the example of a zinc complex by reaction of the title compound as ligand, see: Zhao *et al.* (2004 ▶).
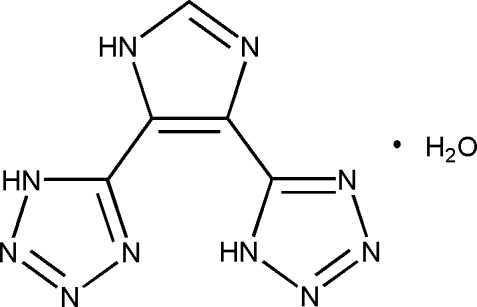


## Experimental

### 

#### Crystal data

C_5_H_4_N_10_·H_2_O
*M* *_r_* = 222.20
Monoclinic, 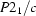

*a* = 15.607 (3) Å
*b* = 3.6706 (7) Å
*c* = 18.127 (7) Å
β = 119.13 (2)°
*V* = 907.1 (5) Å^3^
*Z* = 4
Mo *K*α radiation
μ = 0.13 mm^−1^
*T* = 294 K
0.08 × 0.08 × 0.03 mm

#### Data collection

Rigaku SCXmini diffractometer
Absorption correction: multi-scan (*CrystalClear*; Rigaku, 2005 ▶) *T*_min_ = 0.892, *T*_max_ = 0.990
7767 measured reflections
1785 independent reflections
1362 reflections with *I* > 2σ(*I*)
*R*_int_ = 0.061

#### Refinement

*R*[*F* ^2^ > 2σ(*F* ^2^)] = 0.071
*wR*(*F* ^2^) = 0.201
*S* = 1.06
1785 reflections
153 parameters
H atoms treated by a mixture of independent and constrained refinement
Δρ_max_ = 0.60 e Å^−3^
Δρ_min_ = −0.56 e Å^−3^

### 

Data collection: *CrystalClear* (Rigaku, 2005 ▶); cell refinement: *CrystalClear*; data reduction: *CrystalClear*; program(s) used to solve structure: *SHELXS97* (Sheldrick, 2008 ▶); program(s) used to refine structure: *SHELXL97* (Sheldrick, 2008 ▶); molecular graphics: *SHELXTL* (Sheldrick, 2008 ▶); software used to prepare material for publication: *SHELXTL*.

## Supplementary Material

Crystal structure: contains datablocks I, global. DOI: 10.1107/S1600536809017899/im2114sup1.cif

Structure factors: contains datablocks I. DOI: 10.1107/S1600536809017899/im2114Isup2.hkl

Additional supplementary materials:  crystallographic information; 3D view; checkCIF report

## Figures and Tables

**Table 1:** Hydrogen-bond geometry (Å, °)

*D*—H⋯*A*	*D*—H	H⋯*A*	*D*⋯*A*	*D*—H⋯*A*
O1*W*—H2*W*⋯N3^i^	0.91 (2)	2.12 (2)	3.014 (4)	169 (5)
O1*W*—H1*W*⋯N9^ii^	0.91 (2)	1.99 (2)	2.884 (4)	169 (5)
N2—H2*A*⋯O1*W*^i^	0.86	2.41	3.188 (4)	151
N7—H7*A*⋯N1^iii^	0.86	2.10	2.799 (4)	139
N6—H6*A*⋯N10	0.86	1.95	2.711 (4)	146

Symmetry codes: (i) 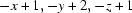; (ii) 
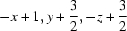
; (iii) 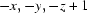.

**Fig. 1.**
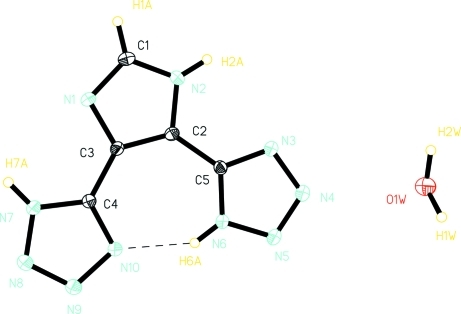
The molecular structure of the title compound showing the atomic numbering scheme. Displacement ellipsoids are drawn at the 30% probability level.

**Fig. 2.**
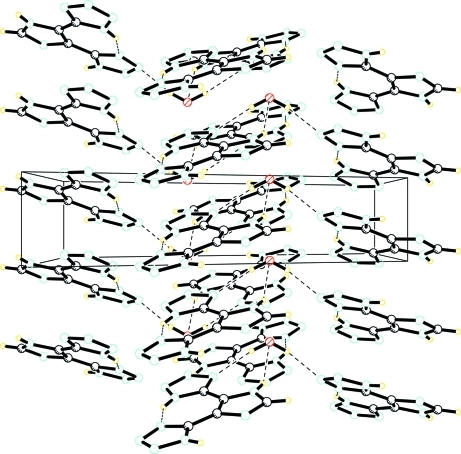
Three-dimensional network of the title compound viewed along a axis.

## supplementary crystallographic information

### Comment

The crystal data show that in the title compound, C_5_H_4_N_10_ × H_2_O, the molecule is essentially planar with dihedral angles between imidazole and the tetrazole rings of 3.5 (2)° and 3.0 (2)°, respectively.

Intramolecular hydrogen bonds between the tetrazole rings determine the conformation of the molecule. It is also interesting to note that strong intermolecular have been found between the tetrazole and imidazole rings towards the solvent water molecules. This results in the formation of a three-dimensional network, as shown in Figure 2.

### Experimental

NaN_3_ (0.975 g,15 mmol) and NH_4_Cl (0.587 g, 11 mmol) were added to a solution of (4,5-Dicyano)-imidazole (1.18 g,10 mmol) in DMF (25 ml) under magnetic stirring in an oil bath. The resulting mixture was heated to 90°C for 8 h until the starting material was fully consumed as shown with the help of TLC detection. The mixture was allowed to cool to room temperature and acidified to pH = 2 with 1*M* aqueous HCl. The resulting precipitate was collected, washed with a small amount of water and dried at 60°C for 12 h. Colorless crystals of the title compound suitable for X-ray diffraction were obtained from an ethanolic solution after one week.

### Refinement

Positional parameters of all the H atoms bonded to C and N atoms were calculated geometrically with *U*_iso_(H) = 1.2*U*_eq_(C,*N*). The O—H hydrogen atoms of the water molecule were located in a difference Fourier map and refined freely with isotropic temperature factors.

### Crystal data

**Table:**

C_5_H_4_N_10_·H_2_O	*F*(000) = 456
*M**_r_* = 222.20	*D*_x_ = 1.627 Mg m^−^^3^
Monoclinic, *P*2_1_/*c*	Mo *K*α radiation, λ = 0.71073 Å
Hall symbol: -P 2ybc	Cell parameters from 2076 reflections
*a* = 15.607 (3) Å	θ = 2.0–27.5°
*b* = 3.6706 (7) Å	µ = 0.13 mm^−^^1^
*c* = 18.127 (7) Å	*T* = 294 K
β = 119.13 (2)°	Block, colorless
*V* = 907.1 (5) Å^3^	0.08 × 0.08 × 0.03 mm
*Z* = 4	

### Data collection

**Table:**

Rigaku SCXmini diffractometer	1785 independent reflections
Radiation source: fine-focus sealed tube	1362 reflections with *I* > 2σ(*I*)
graphite	*R*_int_ = 0.061
Detector resolution: 13.6612 pixels mm^-1^	θ_max_ = 26.0°, θ_min_ = 3.6°
ω scans	*h* = −19→19
Absorption correction: multi-scan (*CrystalClear*; Rigaku, 2005)	*k* = −4→4
*T*_min_ = 0.892, *T*_max_ = 0.990	*l* = −22→22
7767 measured reflections	

### Refinement

**Table:**

Refinement on *F*^2^	Primary atom site location: structure-invariant direct methods
Least-squares matrix: full	Secondary atom site location: difference Fourier map
*R*[*F*^2^ > 2σ(*F*^2^)] = 0.071	Hydrogen site location: inferred from neighbouring sites
*wR*(*F*^2^) = 0.201	H atoms treated by a mixture of independent and constrained refinement
*S* = 1.06	*w* = 1/[σ^2^(*F*_o_^2^) + (0.0998*P*)^2^ + 1.6707*P*] where *P* = (*F*_o_^2^ + 2*F*_c_^2^)/3
1785 reflections	(Δ/σ)_max_ < 0.001
153 parameters	Δρ_max_ = 0.60 e Å^−^^3^
0 restraints	Δρ_min_ = −0.56 e Å^−^^3^

### Special details

**Table:**

Geometry. All e.s.d.'s (except the e.s.d. in the dihedral angle between two l.s. planes) are estimated using the full covariance matrix. The cell e.s.d.'s are taken into account individually in the estimation of e.s.d.'s in distances, angles and torsion angles; correlations between e.s.d.'s in cell parameters are only used when they are defined by crystal symmetry. An approximate (isotropic) treatment of cell e.s.d.'s is used for estimating e.s.d.'s involving l.s. planes.
Refinement. Refinement of *F*^2^ against ALL reflections. The weighted *R*-factor *wR* and goodness of fit *S* are based on *F*^2^, conventional *R*-factors *R* are based on *F*, with *F* set to zero for negative *F*^2^. The threshold expression of *F*^2^ > σ(*F*^2^) is used only for calculating *R*-factors(gt) *etc*. and is not relevant to the choice of reflections for refinement. *R*-factors based on *F*^2^ are statistically about twice as large as those based on *F*, and *R*- factors based on ALL data will be even larger.

### Fractional atomic coordinates and isotropic or equivalent isotropic displacement parameters (Å^2^)

**Table:**

	*x*	*y*	*z*	*U*_iso_*/*U*_eq_	
O1W	0.6668 (2)	1.0057 (9)	0.61964 (19)	0.0429 (8)	
N5	0.4974 (2)	0.4374 (9)	0.67749 (19)	0.0313 (8)	
N6	0.40302 (19)	0.3471 (9)	0.64859 (17)	0.0260 (7)	
H6A	0.3794	0.2519	0.6783	0.031*	
N9	0.2305 (2)	−0.0391 (9)	0.73314 (18)	0.0305 (7)	
N10	0.2612 (2)	0.0904 (9)	0.68015 (18)	0.0267 (7)	
N1	0.0913 (2)	0.2414 (9)	0.45237 (17)	0.0270 (7)	
N7	0.1017 (2)	0.0035 (9)	0.61097 (18)	0.0302 (8)	
H7A	0.0420	−0.0063	0.5709	0.036*	
N8	0.1358 (2)	−0.0896 (10)	0.69193 (19)	0.0337 (8)	
N4	0.5023 (2)	0.5693 (9)	0.61343 (19)	0.0294 (7)	
N2	0.2075 (2)	0.4482 (8)	0.42653 (17)	0.0263 (7)	
H2A	0.2371	0.5317	0.4006	0.032*	
N3	0.4130 (2)	0.5680 (8)	0.54265 (17)	0.0267 (7)	
C1	0.1142 (3)	0.3667 (11)	0.3940 (2)	0.0314 (9)	
H1A	0.0688	0.3924	0.3371	0.038*	
C5	0.3516 (2)	0.4283 (9)	0.5666 (2)	0.0201 (7)	
C4	0.1794 (2)	0.1138 (9)	0.6055 (2)	0.0212 (7)	
C2	0.2476 (2)	0.3706 (9)	0.5112 (2)	0.0209 (7)	
C3	0.1767 (2)	0.2407 (9)	0.5288 (2)	0.0211 (7)	
H1W	0.698 (3)	1.123 (14)	0.670 (2)	0.067 (16)*	
H2W	0.635 (3)	1.117 (14)	0.568 (2)	0.069 (17)*	

### Atomic displacement parameters (Å^2^)

**Table:**

	*U*^11^	*U*^22^	*U*^33^	*U*^12^	*U*^13^	*U*^23^
O1W	0.0462 (18)	0.0463 (18)	0.0335 (17)	−0.0038 (14)	0.0172 (15)	0.0008 (14)
N5	0.0211 (15)	0.044 (2)	0.0262 (16)	−0.0073 (13)	0.0094 (13)	−0.0022 (14)
N6	0.0198 (14)	0.0368 (17)	0.0210 (14)	−0.0051 (13)	0.0095 (12)	0.0011 (12)
N9	0.0284 (16)	0.0370 (18)	0.0242 (15)	−0.0031 (13)	0.0114 (13)	0.0054 (13)
N10	0.0215 (14)	0.0366 (17)	0.0201 (14)	−0.0032 (13)	0.0088 (12)	0.0036 (13)
N1	0.0198 (14)	0.0370 (18)	0.0192 (14)	−0.0032 (13)	0.0055 (12)	0.0010 (13)
N7	0.0207 (15)	0.0437 (19)	0.0223 (15)	−0.0063 (13)	0.0074 (12)	0.0039 (13)
N8	0.0302 (17)	0.046 (2)	0.0254 (16)	−0.0057 (15)	0.0142 (14)	0.0056 (14)
N4	0.0236 (15)	0.0361 (18)	0.0273 (16)	−0.0066 (13)	0.0114 (13)	−0.0014 (13)
N2	0.0244 (15)	0.0352 (17)	0.0211 (15)	−0.0028 (13)	0.0125 (13)	0.0034 (13)
N3	0.0209 (14)	0.0338 (17)	0.0238 (15)	−0.0041 (13)	0.0097 (13)	0.0004 (13)
C1	0.0250 (18)	0.045 (2)	0.0185 (17)	−0.0020 (16)	0.0058 (15)	0.0032 (15)
C5	0.0208 (16)	0.0219 (16)	0.0199 (16)	−0.0018 (13)	0.0117 (14)	−0.0015 (13)
C4	0.0199 (16)	0.0236 (17)	0.0192 (16)	−0.0014 (14)	0.0087 (13)	0.0008 (13)
C2	0.0206 (16)	0.0222 (17)	0.0177 (16)	−0.0023 (13)	0.0076 (13)	−0.0028 (13)
C3	0.0168 (15)	0.0263 (18)	0.0193 (16)	0.0016 (13)	0.0081 (13)	0.0003 (14)

### Geometric parameters (Å, °)

**Table:**

O1W—H1W	0.91 (2)	N7—N8	1.339 (4)
O1W—H2W	0.91 (2)	N7—H7A	0.8600
N5—N4	1.294 (4)	N4—N3	1.359 (4)
N5—N6	1.343 (4)	N2—C1	1.313 (4)
N6—C5	1.335 (4)	N2—C2	1.375 (4)
N6—H6A	0.8600	N2—H2A	0.8600
N9—N8	1.303 (4)	N3—C5	1.332 (4)
N9—N10	1.352 (4)	C1—H1A	0.9300
N10—C4	1.336 (4)	C5—C2	1.450 (4)
N1—C1	1.351 (5)	C4—C3	1.446 (4)
N1—C3	1.378 (4)	C2—C3	1.378 (5)
N7—C4	1.327 (4)		
			
H1W—O1W—H2W	125 (5)	C5—N3—N4	105.3 (3)
N4—N5—N6	105.9 (3)	N2—C1—N1	112.6 (3)
C5—N6—N5	109.3 (3)	N2—C1—H1A	123.7
C5—N6—H6A	125.4	N1—C1—H1A	123.7
N5—N6—H6A	125.4	N3—C5—N6	108.1 (3)
N8—N9—N10	109.7 (3)	N3—C5—C2	124.8 (3)
C4—N10—N9	104.4 (3)	N6—C5—C2	127.1 (3)
C1—N1—C3	107.0 (3)	N7—C4—N10	111.2 (3)
C4—N7—N8	105.6 (3)	N7—C4—C3	124.7 (3)
C4—N7—H7A	127.2	N10—C4—C3	124.1 (3)
N8—N7—H7A	127.2	N2—C2—C3	110.5 (3)
N9—N8—N7	109.1 (3)	N2—C2—C5	119.4 (3)
N5—N4—N3	111.4 (3)	C3—C2—C5	130.1 (3)
C1—N2—C2	104.8 (3)	C2—C3—N1	105.0 (3)
C1—N2—H2A	127.6	C2—C3—C4	133.1 (3)
C2—N2—H2A	127.6	N1—C3—C4	121.9 (3)
			
N4—N5—N6—C5	−0.3 (4)	C1—N2—C2—C3	0.1 (4)
N8—N9—N10—C4	0.1 (4)	C1—N2—C2—C5	179.6 (3)
N10—N9—N8—N7	0.2 (4)	N3—C5—C2—N2	2.9 (5)
C4—N7—N8—N9	−0.4 (4)	N6—C5—C2—N2	−175.6 (3)
N6—N5—N4—N3	0.0 (4)	N3—C5—C2—C3	−177.6 (4)
N5—N4—N3—C5	0.3 (4)	N6—C5—C2—C3	3.8 (6)
C2—N2—C1—N1	0.1 (4)	N2—C2—C3—N1	−0.2 (4)
C3—N1—C1—N2	−0.2 (5)	C5—C2—C3—N1	−179.6 (3)
N4—N3—C5—N6	−0.5 (4)	N2—C2—C3—C4	178.4 (3)
N4—N3—C5—C2	−179.2 (3)	C5—C2—C3—C4	−1.1 (6)
N5—N6—C5—N3	0.5 (4)	C1—N1—C3—C2	0.2 (4)
N5—N6—C5—C2	179.2 (3)	C1—N1—C3—C4	−178.6 (3)
N8—N7—C4—N10	0.5 (4)	N7—C4—C3—C2	178.5 (4)
N8—N7—C4—C3	−179.9 (3)	N10—C4—C3—C2	−1.9 (6)
N9—N10—C4—N7	−0.3 (4)	N7—C4—C3—N1	−3.1 (5)
N9—N10—C4—C3	−179.9 (3)	N10—C4—C3—N1	176.4 (3)

### Hydrogen-bond geometry (Å, °)

**Table:**

*D*—H···*A*	*D*—H	H···*A*	*D*···*A*	*D*—H···*A*
O1W—H2W···N3^i^	0.91 (2)	2.12 (2)	3.014 (4)	169 (5)
N2—H2A···O1W^i^	0.86	2.41	3.188 (4)	151
O1W—H1W···N9^ii^	0.91 (2)	1.99 (2)	2.884 (4)	169 (5)
N7—H7A···N1^iii^	0.86	2.10	2.799 (4)	139
N6—H6A···N10	0.86	1.95	2.711 (4)	146

Symmetry codes: (i) −*x*+1, −*y*+2, −*z*+1; (ii) −*x*+1, *y*+3/2, −*z*+3/2; (iii) −*x*, −*y*, −*z*+1.

## References

[bb1] Rigaku (2005). *CrystalClear* Rigaku Corporation, Tokyo, Japan.

[bb2] Sheldrick, G. M. (2008). *Acta Cryst.* A**64**, 112–122. 10.1107/S0108767307043930 18156677

[bb3] Zhao, H., Ye, Q., Wu, Q., Song, Y.-M., Liu, Y.-J. & Xiong, R.-G. (2004). *Z. Anorg. Allg. Chem.* **630**, 1367–1370.

